# *Norpa* Signalling and the Seasonal Circadian Locomotor Phenotype in *Drosophila*

**DOI:** 10.3390/biology9060130

**Published:** 2020-06-16

**Authors:** Carlo Breda, Ezio Rosato, Charalambos P. Kyriacou

**Affiliations:** Department of Genetics and Genome Biology, University of Leicester, Leicester LE1 7RH, UK; carlo.breda@dmu.ac.uk (C.B.); er6@leicester.ac.uk (E.R.)

**Keywords:** *Drosophila*, circadian, seasonal, locomotor, *norpA*, *per 3′ UTR*, *plc21c*

## Abstract

In this paper, we review the role of the *norpA*-encoded phospholipase C in light and thermal entrainment of the circadian clock in *Drosophila melanogaster.* We extend our discussion to the role of *norpA* in the thermo-sensitive splicing of the *per 3′ UTR*, which has significant implications for seasonal adaptations of circadian behaviour. We use the *norpA* mutant-generated enhancement of *per* splicing and the corresponding advance that it produces in the morning (M) and evening (E) locomotor component to dissect out the neurons that are contributing to this *norpA* phenotype using GAL4/UAS. We initially confirmed, by immunocytochemistry and *in situ* hybridisation in adult brains, that *norpA* expression is mostly concentrated in the eyes, but we were unable to unequivocally reveal *norpA* expression in the canonical clock cells using these methods. In larval brains, we did see some evidence for co-expression of NORPA with PDF in clock neurons. Nevertheless, downregulation of *norpA* in clock neurons did generate behavioural advances in adults, with the eyes playing a significant role in the *norpA* seasonal phenotype at high temperatures, whereas the more dorsally located CRYPTOCHROME-positive clock neurons are the likely candidates for generating the *norpA* behavioural effects in the cold. We further show that knockdown of the related *plc21C* encoded phospholipase in clock neurons does not alter *per* splicing nor generate any of the behavioural advances seen with *norpA.* Our results with downregulating *norpA* and *plc21C* implicate the rhodopsins Rh2/Rh3/Rh4 in the eyes as mediating *per 3′ UTR* splicing at higher temperatures and indicate that the CRY-positive LNds, also known as ‘evening’ cells are likely mediating the low-temperature seasonal effects on behaviour via altering *per 3′UTR* splicing.

## 1. Introduction

The circadian clock regulates the 24 h cycles of behaviour, physiology and metabolism of animals and plants as well as some bacteria that live on the surface of our planet [1]. These cycles have evolved because of the selection pressure that is applied by the relentless 24 h rotation of the Earth around its axis. Consequently, circadian clocks have fitness value, presumably because they allow organisms to anticipate the regularly changing cycles of light and dark, warmth and cold [2,3,4,5]. The Earth’s tilt around its axis also generates the annual cycles of winter and summer in the northern and southern hemispheres during its 365 day orbit around the Sun. Organisms have also adapted to these seasonal cycles with complex behavioural and metabolic phenotypes such as migration and hibernation.

Konopka and Benzer laid the foundations for the subsequent molecular analysis of the circadian clock in *Drosophila* by their ground-breaking mutagenesis that delivered the classic *period (per)* mutations [6]. Identifying the DNA sequences that encoded *period* by transgenic rescue of the arrhythmic locomotor phenotype of the null mutant *per*^01^ by the groups of Jeff Hall and Michael Rosbash at Brandeis University and Mike Young at Rockefeller heralded the dawn of ‘molecular neurogenetics’ and the discovery of the negative feedback loop that underlies clock function [7,8,9]. The entry of many other research groups into this field, in *Drosophila* and then mammals [10], revealed that the molecular clockwork was largely conserved in these higher eukaryotes and was to lead to the award of the Nobel Prize for Medicine or Physiology to Hall, Rosbash and Young in 2017 [11].

The fly clock consists of a negative feedback loop in which two negative autoregulators encoded by the *per* and *timeless (tim)* genes are rhythmically transcribed. The cycle begins when, late in the day, two bHLH transcription factors CLOCK (CLK) and CYCLE (CYC) bind regulatory regions known as E-boxes (*CACGTG*) on the *per* and *tim* promoters [12]. Then, after transcription and translation, PER and TIM undergo a series of post-translational modifications, which delay their accumulation and nuclear translocation. Late in the night, PER and TIM are at their peak, and they enter the nucleus and sequester the CLK–CYC dimer, shutting down their own transcription. Finally, with the start of the day, PER and TIM degrade via further post-translational modifications that differ under light–dark (LD) or constant darkness (DD) conditions. CLK–CYC returns to the *per/tim* promoters and the transcription–translation cycle begins again. Two other loops intersect with the PER–TIM cycle—one involving PDP1ε and VRILLE and another involving CLOCKWORK ORANGE (CWO), seemingly to stabilise the system [12].

Although all the above occurs even under constant darkness, in a more realistic light–dark environment, the blue light-sensitive protein CRYPTOCHROME (CRY) intervenes. CRY is activated by light, so its conformation changes at dawn and the active form binds TIM. This interaction is mediated by the E3 ubiquitin ligases JETLAG and RAMSHACKLE [13,14,15,16], resulting in the degradation of TIM and consequently of PER [17].

In the fly, the canonical clock genes are expressed in many tissues [18] but robust rhythmic locomotor behaviour (a phenotype easily measured in the laboratory) requires expression in approximately 150 neurons in the brain (~75 per hemisphere) that are mainly divided into three groups of later neurons (LNs) and three groups of dorsal neurons (DNs). The LNs comprise the ventral groups, small (s-LNvs, four cells) and large (l-LNvs, four cells), both producing the neuropeptide pigment-dispersing factor (PDF), and the dorsal group (LNds, six cells). An additional, single 5th-LNvs producing the ion transport peptide (ITP) is also present. The DNs are divided into three groups, DN1s (~16 cells—2 anterior and ~14 posterior), DN2s (two cells) and DN3s (~40 cells) (reviewed in [12]). Finally, a group of lateral posterior neurons (LPN, three cells) has been investigated recently in the context of circadian and sleep cycles [19]. Historically, some clock neurons have been considered prominent in generating the major features of locomotor behaviour and deemed necessary and sufficient for self-sustained rhythmicity [20,21]. The current evidence suggests that the network is not a hierarchy, but represents a plastic and flexible entourage of neurons that can influence each other to meet different environmental demands [22,23,24,25,26] 

The light input pathway to the clock therefore has CRY at its core, so the neurons in which CRY is expressed—the s-LNvs, l-LNvs (the large ventral neurons also express PDF), three of the six LNds and some of the DN1s (dorsal neurons)—in addition to the eyes, become a focus for light entrainment [27]. The visual photoreceptors are involved because even in a cry-null *(cry*^0^*)* or cry-nearly-null *(cry^b^)* mutant, locomotor behaviour can still be entrained to light–dark cycles although the mutants do not respond to a brief light pulses early or late at night with the customary locomotor phase shifts [28]. These additional visual photoreceptors are found in the eyes, Hofbauer–Buchner (H–B) eyelet and the ocelli, and they express a variety of rhodopsins [29]. There are seven rhodopsins and most of them are expressed in the R1–R8 photoreceptor cells of each ommatidium that make up the compound eye [30,31]. Rhodopsin1 (Rh1/ninaE) is expressed in the R1–R6 outer cells, while Rh3/Rh4 and Rh5/Rh6 are expressed in the two inner cells, R7 and R8, respectively. Rh6 is also expressed in the H–B eyelet, while Rh2 is limited to the ocelli, although a recent study has detected the transcript in R7 [32]. Rh7 has recently been identified and is localised to the sLNvs and may signal through Gq protein alpha subunit and PLC21C [31]. Rhodopsins are G-protein-coupled receptors (GPCRs) with Rh1–Rh6 utilising phospholipase-Cβ (PLC-β in the phototransduction pathway which is encoded by *norpA,* while Rh1/Rh5/Rh6 can also signal to the sLNvs via a *norpA*-independent pathway [33].

*norpA^P^*^4 1^mutants can still synchronise to LD cycles because CRY is present and, under high light intensity even *norpA^P^*^41^, *cry^b^* flies can slowly entrain [33]. Notably, adding mutations of *Rh1*, *Rh5* and *Rh6* blocks entrainment, the explanation being that Rh1, Rh5 and Rh6 signal via Gq and PLC21C [33]. This implicates the R1–R6 and R8 photoreceptor cells and the H–B eyelet in a *norpA*-independent light response that causes the degradation of TIM (and consequently PER) in the s-LNvs [33]. Rh7 is also coupled to PLC21C and *Rh7* mutants reveal defects in circadian locomotor responses to brief light pulses. Yet, *Rh7* mutations do not obliterate the synchronisation to LD cycles of the *norpA^P^*^41^*; cry^b^* double mutants as do *Rh1*/*Rh5*/*Rh6* [31,33]. One possible caveat here is that Rh7 appears to be more sensitive to blue light and the white-light LEDs used in the triple mutant experiments may not have optimally engaged the Rh7 photoreceptor molecule [33].

While light is considered the strongest Zeitgeber, for poikilotherms such as Drosophila, environmental temperature cycles might also be expected to play an important role in the entrainment of circadian behaviour, particularly as *D. melanogaster* is found in both tropical and temperate regions of the world [34]. Indeed, under natural conditions such as the hot summers of continental Europe, locomotor activity shows a very different profile to the simple morning and evening peaks that are separated by the ‘siesta’ and are observed under standard laboratory conditions of constant warm temperature in rectangular LD cycles. An additional afternoon or ‘A’ locomotor peak is seen under these natural conditions, which can be much more prominent than the M and E components, particularly under open-field conditions [35,36]. This A peak is regulated by the heat-sensitive TrpA1 channel and reflects a stress response of flies to the higher summer temperatures that would be common in temperate regions of Europe and the Americas [36,37].

However, under laboratory conditions, low-amplitude temperature cycles as small as 2 °C are able to synchronise the fly clock [38]. Temperature cycles can even restore molecular cycling of PER and TIM in LL, which would otherwise be arrhythmic at constant temperature, so, somehow, the superimposed thermal rhythms must suppress the normal CRY-mediated TIM and PER degradation that would be expected to occur under constant light [39]. A possible target could be the glutamate signalling pathway from the DNs to the s-LNvs, which, when disrupted, generates rhythms in LL [40]. Screening for temperature entrainment defects identified the *nocte* mutant, which was neither able to synchronise its locomotor behaviour nor *per-luciferase* (*per-luc*) reporter cycling to a warm-cold thermal cycle [39]. *nocte* encodes a glutamine-rich protein with poly-Q and poly-A repeats, but its function is unknown [41]. Somewhat surprisingly, *norpA* mutants also showed a similar defect in temperature entrainment so PLC-β plays a role in both thermal and photoentrainment [39]. Under LL, the amplitude of the cycling of PER and TIM, which is restored with temperature cycles, is significantly reduced in *nocte* mutants [39].

Isolated peripheral organs can synchronise their *per-luc* reporter cycles to temperature cycles, whereas the isolated brain cannot; thus, the brain must receive thermal signals from the periphery [41]. It was therefore of considerable interest when downregulation of *nocte* in the periphery produced defective temperature entrainment and expression studies revealed *nocte* to be expressed in chordotonal organs which act a stretch receptors in limb joints, in addition to other external sensory organs. The chordotonal organs do not appear to contain a clock so *nocte* is not interfering with any circadian function in the periphery [41]. Rather *nocte* mutants are disrupting the temperature signals from these organs to the brain, particularly to the more dorsal group of clock neurons, DN1–3s and LNds [42]. In fact, the mutants also reveal defects in chordotonal anatomy. As these sensory organs are mechanosensors, cycling vibrational stimuli are also sufficient to entrain circadian locomotor behaviour [43]. Furthermore, the ionotropic receptor 25a (IR25a) is also expressed in chordotonal organs and can mediate the temperature entrainment of locomotor rhythms and molecular cycles in the DN1 and LNd clock neurons to very low amplitude temperature oscillations [44]. Thus, the mechanosensory system plays an important role in conveying temperature information to the brain clock, particularly the dorsal neurons, as well as, presumably, proprioreceptive feedback from the fly’s own activity. Given that natural temperature cycles show a slow change during the day, it is likely that the effects observed in the laboratory that are usually driven under square-wave temperature cycles with sudden ‘on–off’ switches every 12 h underestimate the importance of this input [35].

The *nocte* and *norpA* genes are therefore key players in the daily circadian light and temperature entrainment to thermal cycles [39,42]. However, *D. melanogaster* are cosmopolitan, so they face dramatic seasonal changes in temperate regions. What happens under colder or warmer conditions? One mechanism that adapts the clock to temperature change has been elegantly dissected by the group of Isaac Edery and involves the splicing of a *per* 3′ intron. Under colder conditions, splicing is upregulated with the result that the *per* transcript and protein cycles are advanced, leading to an advance in the main evening component of locomotor behaviour [45]. This can be seen as an adaptive response in that under colder conditions the evening component of activity allows the animal to forage during the warmer times of the day. Under warmer temperatures, *per* 3′ splicing is reduced with a corresponding delay in *per* product cycling and a concomitant delay in evening activity to the light-off signal. This allows the fly to avoid desiccation during the hottest parts of the day by extending its mid-afternoon ‘siesta’. While we have already discussed the thermal stress response mediated by *TrpA1* that generates a large afternoon component under natural conditions and which overrides the normal siesta [36], the *per* splicing phenomenon is intriguing in that it depends upon low-affinity splice sites which become more functional under cold temperatures [46]. An exclusively tropical species such as *Drosophila yakuba*, has high affinity splice sites so that *per* splicing does not change between high and low temperatures, nor does the locomotor profile [46]. As photoperiodic and thermal seasonal changes are minimal at the equator, this would also appear to provide an adaptive response for tropical species.

The splicing of the *per 3*′ *UTR* can also be interpreted as generating alterations in sleep rather than changes in locomotor activity, so that under warmer conditions, arousal is reduced and the flies sleep longer during the siesta thereby delaying the upward swing of the E locomotor component [47]. Natural polymorphisms in the *per 3*′ *UTR* can modulate the properties of the siesta [48] and an altitudinal cline has been observed in African populations in which individuals at higher altitudes had reduced siestas [49]. This correlated with a particular single-nucleotide variant in the *per 3*′ *UTR* that altered splicing efficiency. Sequence variation in the *per 3*′ *UTR* has also been implicated in adaptation of the mid-day siesta in tropical versus temperate regions [50].

Splicing of the *per 3*′ *UTR* can be enhanced by *norpA* mutations [51,52]. Consequently, in the null mutant, *norpA^P^*^41^, the increased *per* splicing leads to an even larger advance in the evening component of locomotor activity under both warmer and colder conditions compared to the wild type [51,52]. In fact, the locomotor activity peak of the wild type under cold conditions corresponds to the *norpA^P^*^41^ peak under warmer temperatures so the mutant behaves as if it is colder that it actually is [51]. NORPA is expressed predominantly in the eyes and the ocelli and in several other tissues including the brain where it is unlikely to serve a photoreceptive function, suggesting that the PLC-β is used in other signal transduction pathways [53,54,55,56]. However, the effect of *norpA* mutation on *per* splicing and the advance in the evening peak of locomotor behaviour, which is the most prominent phenotype associated with the mutation, has not been anatomically mapped. Given that *norpA*’s reported highest levels of expression are in the eyes, these might represent the tissues that are driving the mutant’s enhanced *per 3*′ *UTR* splicing and corresponding behavioural responses.

We have therefore used the effects of the *norpA* mutation on *per* splicing as a tool for identifying the neuronal basis of the seasonal response. This requires some prior knowledge of *norpA* expression patterns in the adult brain. The spatial localisation studies cited above used tissue sections, so we decided to perform further localisation of NORPA/*norpA* in whole mount fly brain, both adult and larval, counterstained with reagents against circadian clock gene products to provide a more accurate spatial representation in relation to clock neurons. We therefore scrutinised the spatial localisation of *norpA* before performing a *Gal4/UAS* anatomical dissection of the ‘seasonal neurons’.

## 2. Methods

*D. melanogaster* were maintained at either 18 or 25 °C under a cycle of 12 h of light/dark (LD 12:12). The *Gal4* lines used for the locomotor studies were *gmrGal4*, *timGal4*, *PdfGal4*, *timGal4cryGal80*, *mai179Gal4*, *clk6-1Gal4* (gift from Dr. Nick Glossop) and *clk6-1Gal4; cryGal80* and *R32* (an enhancer trap line inserted in *clockwork-orange*, *cwo* that expresses *lacZ* in clock neurons) have been described previously [57,58]. The experimental flies were crossed to *UASnorpA or UASplc21C* RNAi lines (VDRC lines 21490 and 25558, respectively), whereas the control flies were represented by the *Gal4/Gal80* lines or the *UASRNAi* lines crossed to *w*^1118^. Locomotor activity rhythms (from male flies) were recorded at 18 and 29 °C with DAM2 (Trikinetics, Waltham, MA, USA) monitors as described previously [35]. Locomotor activity was recorded in 30 min time bins over 7–12 days in LD12:12 and analysed using Excel (Microsoft, Redmond, WA, USA). For each 24 h cycle we took the time bin with the peak level of evening activity and then found the time bin preceding that bin which contained 50% or more of the evening peak activity value (EZt50). The same process was repeated to find the corresponding morning value (MZt50), thereby generating phase markers for both morning and evening locomotor components. For each fly we averaged these M and E phase markers across the several days of activity monitoring producing a Mean MZt50 and EZt50. This method allows us to normalise the levels of activity among different genotypes, although we present the raw mean activity counts in the figures. Under 18 °C, we observed that the first bin of the day (Zt24.5 or Zt0.5) always had the highest activity counts and also corresponded to the MZt50 because activity levels ramp up very quickly, so MZt50 was not informative. At 29 °C, the final bin of the day or the next bin always had the peak activity levels as this reflects the warmer temperature pushing the evening locomotor peak to the end of the day so the EZt50 values are more informative than the peak values. The Zt50 data were not normally distributed so we used non-parametric Kruskal–Wallis one way ANOVA followed by Dunn’s post-hoc tests, as implemented in the Statistica package (Statsoft, Tulsa, OK, USA). Unless the experimental flies’ values fell at intermediate values to the parental controls, only significant differences between the downregulated genotype and both controls were considered to be of further interest.

Brain dissection and immunofluorescence were carried out as described previously [59]. Details of all the antibodies used are provided in Supplementary methods. The in situ protocol and labelling of RNA probes have been described previously and are further documented in Supplementary Methods [60]. The RNAi lines for *norpA* and *plc21C* downregulation were also assessed for the level of knockdown using Western blots, respectively, at 18 and 29 °C by driving with *actin-GAL4* (All details in Supplementary Materials).

## 3. Results

### 3.1. α-NORPA Antibody Does Not Penetrate into the Deeper Layers of the Adult CNS but Labels Neurons in the Larval Brain

**Figure 1:**
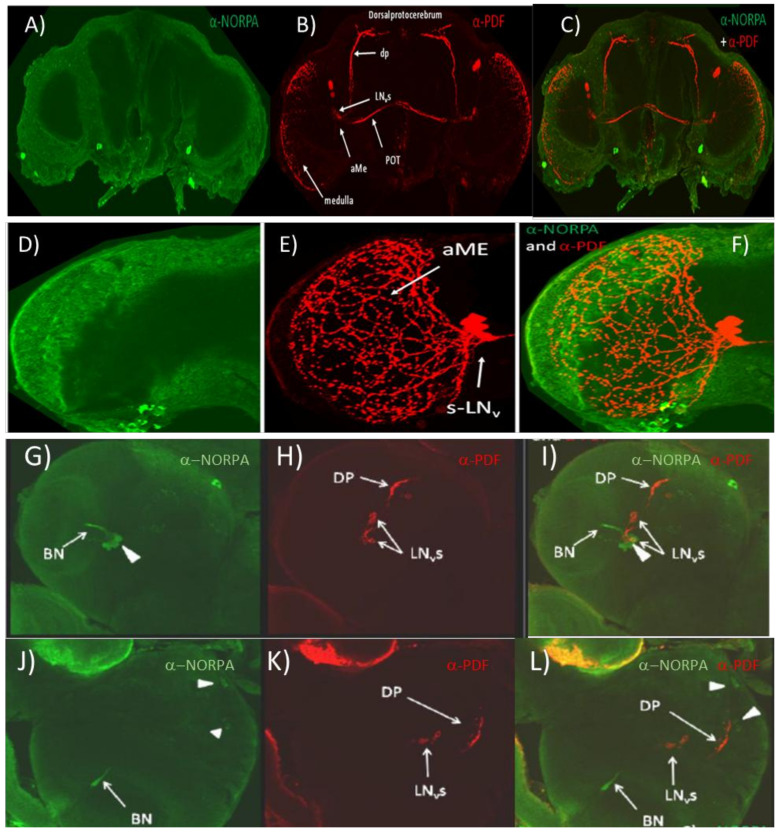
NORPA and PDF expression in adult and larval brains (**A**–**C**). *Drosophila* adult brain in which NORPA was overexpressed in the PDF neurons via *Pdfgal4.* (**A**) α-NORPA signal (green), (**B**) α-PDF signal (red), and (**C**) Merge of images (**A**) and (**B**). (**D**–**F**). Enlargement of an optic lobe overexpressing NORPA in PDF neurons. (**D**) α-NORPA (green), (**E**) α-PDF (red), and (**F**) Merged of (**D**) and (**E**) signals. (**G**–**I**). Ventral view of 3rd instar brain of *w^1118^* larva. (**G**) α-NORPA (green), (**H**) α-PDF (red), and (**I**) merge. BN, Bolwig neuron; DP, dorsal projection of LNvs. Arrowhead on panel I shows LNv neuron that may be co-expressing both NORPA and PDF. (**J**–**L**). Dorsal view of 3rd instar brain of *w*^1118^ larva. (**J**) α-NORPA (green), (**K**) α-PDF (red), and (**L**) merge. Arrowheads in panel L show putative α-NORPA staining in DNs.

Figure S1 shows *R32* transgenic *Drosophila* brains expressing lacZ in circadian neurons and labelled with α-LACZ and α-NORPA antibodies. The expression pattern displayed by the α-LACZ (circadian reporter) antibody was specific and localised in all major circadian neurons, s-LNvs, l-LNvs, LNds, as well as most DNs. On the other hand, the expression profile of NORPA appeared to be mostly on the surface of the brain, particularly the optic lobes but with no signal emerging from the inner portions of the brain where the circadian clock cells are located. These results were also confirmed in *w*^1118^ brains (Figure S2A) using α-NORPA and α-PDF antibodies. PDF antigen labelled the LNv neurons (s-LNvs and l-LNvs) but no co-localisation signals were detected, suggesting that NORPA may be not expressed in the circadian clock neurons. We next examined NORPA labelling in *norpA*-null mutants and no specific staining was observed (Figure S2B). Finally, flies overexpressing NORPA in the LNvs (*w*; *PdfGal4*/+; UAS *norpA*/*+*) were studied. NORPA signal was again distributed mostly in the optic lobes with localisation predominantly on the surface of the brain and with no co-labelling of α-NORPA and α-PDF in the LNvs (Figure 1A–C). However, when an enlargement of the optic lobe and LNs was analysed, overlapping signals were observed only in the contact area between the optic lobe surface and the termination of the PDF arborisation (Figure 1D–F). From these results, it is evident that the NORPA antibody was not able to penetrate the adult brain structures, otherwise a signal would be expected in the LNvs when driven by *PdfGal4.*

We therefore attempted to use α-PDF and α-NORPA on 3rd instar larvae to validate whether the latter reagent was working. It has been reported that NORPA is expressed in the Bolwig nerve fibres that may contact the LNvs [61]. We observed a very similar result but there was also a suggestion that some of the LNvs may be coexpressing PDF and NORPA (Figure 1G–I). We also noticed NORPA expression in cells that might represent putative DNs (Figure 1J–L). Consequently, α-NORPA can penetrate the larval but not the adult brain and, given the possible co-expression of NORPA in clock neurons in the larva, it may be that this is also maintained in the adult.

### 3.2. In Situ Hybridisation Reveals Expression of norpA in Clock Neurons

**Figure 2:**
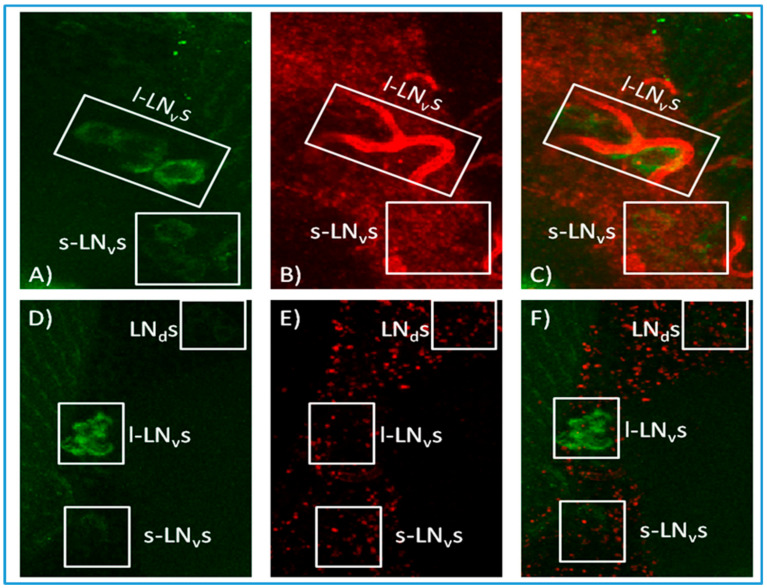
In situ hybridisation. Ventral views of representative R32 adult brains probed with α-LACZ antibody ((**A**,**D**), green), antisense *norpA* probe (concentrated 75 ng, (**B**), red)—the bright red staining around the PDF cells may represent trachea, and sense *norpA* probe (concentrated 150 ng, (**E**), red). (**C**) and (**F**) are a merge of (**A**,**B**) and (**D**,**E**), respectively. The brains presented are images of 10 independent layers merged together.

**Figure 3:**
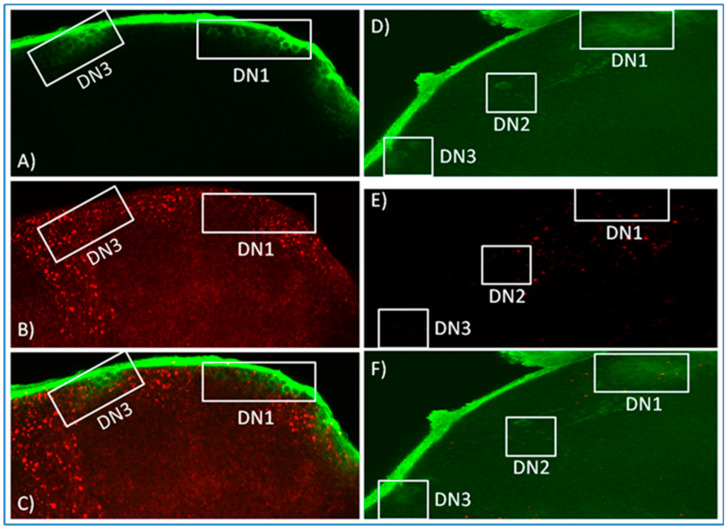
In situ hybridisation. Dorsal views of representative R32 adult brains probed with α-LACZ antibody ((**A**,**D**), green), antisense *norpA* probe (concentrated 75 ng, (**B**), red) and sense *norpA* probe (concentrated 150 ng, (**E**), red). (**C**) and (**F**) are a merge of (**A**,**B**) and (**D**,**E**), respectively. The brains presented are images of 10 independent layers merged together.

We therefore altered our approach and generated antisense *norpA* probes for in situ hybridisation to *R32* brains in order to distinguish among the populations of circadian neurons. In all the layers of the brains analysed, *norpA* was broadly distributed in CNS tissues. Furthermore, when the analysis was focused on the LNvs and DNs, *norpA* signal emerged from the vicinity of these cells (Figure 2 and Figure 3) and sometimes even apparently within the sLNvs but no co-localisation was evident as a merged yellow signal. The *R32* brains were also processed with *norpA* sense probe as a control for verifying the hybridisation efficiency (Figure 2 and Figure 3). Even though the concentration of the control probe was double that of the antisense (150 ng), extremely weak signals were observed and none at the level of the circadian neurons. These results are equivocal and we cannot state with any certainty that *norpA* transcripts and *per* reporter antibody staining co-localise in canonical clock neurons.

### 3.3. Behavioural Effects of Knockdown of norpA in Clock Cells

**Figure 4:**
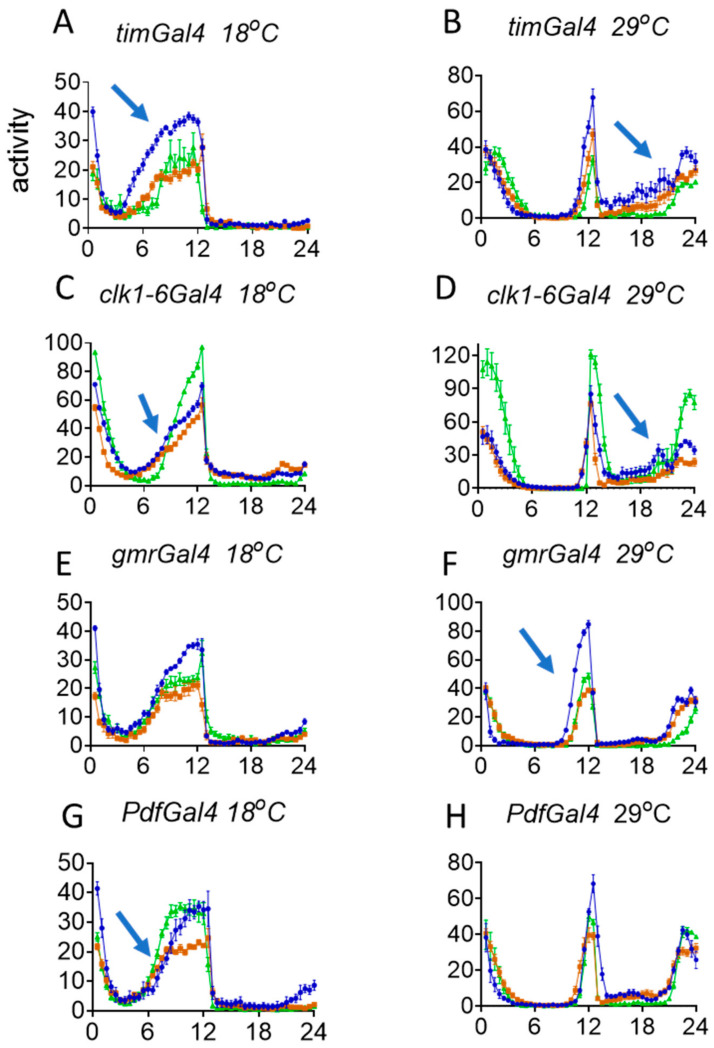

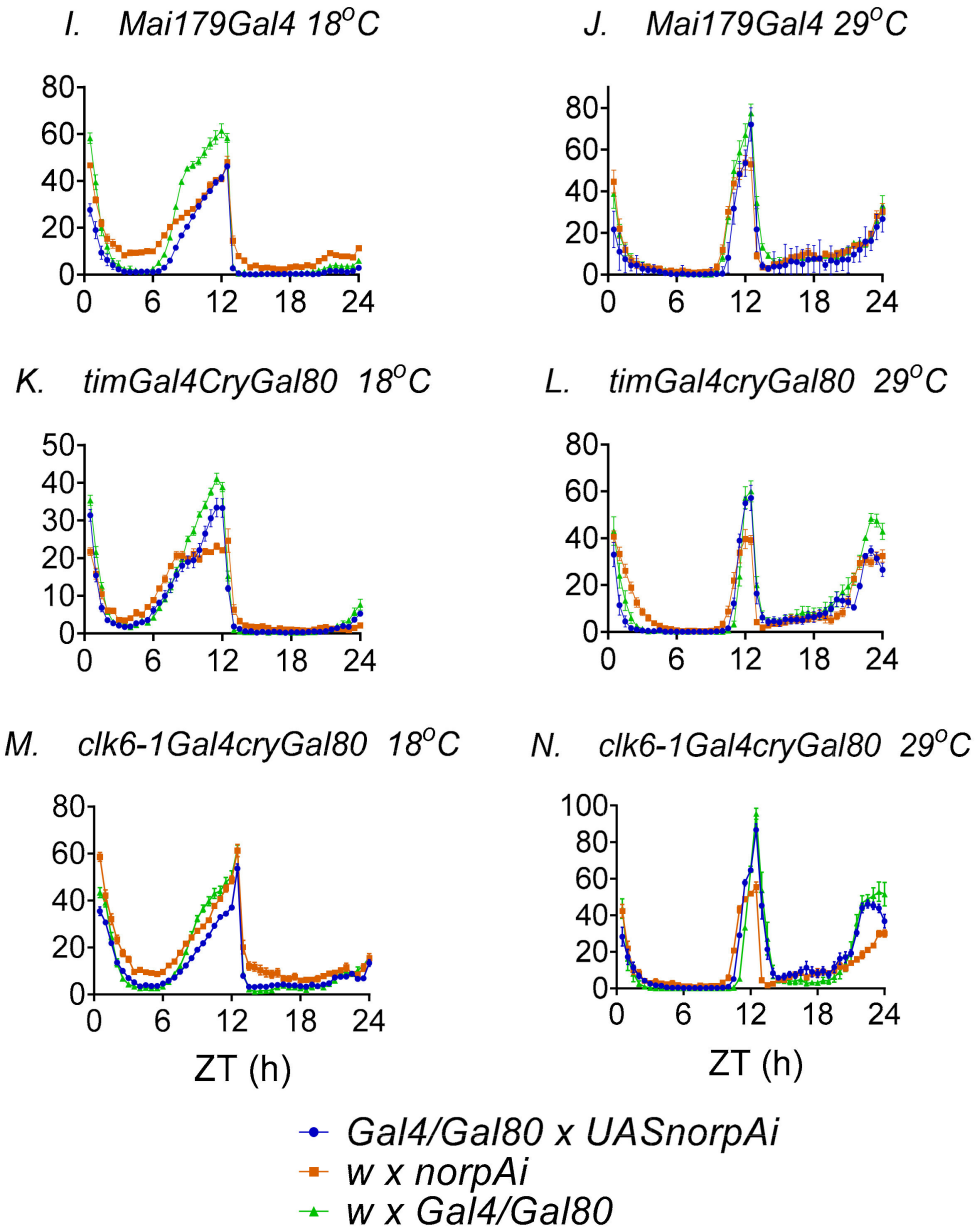
Locomotor activity: *norpA* RNAi in clock cells under different temperatures. *norpA* RNAi and locomotor behaviour. (**A**–**N**) *Gal4/Gal80* Genotypes and temperature given above each panel. *Y* axis, mean locomotor event/30 min time bin. *X* axis, ZT, ZT 0–12 light, ZT 12–24 dark. Blue trace is experimental *Gal4/Gal80 x UASnorpAi*; brown is *w x UASnorpAi*; green is *w x Gal4/Gal80*. Mean +/- sem. Blue arrows show significant effects via Kruskal–Wallis ANOVA and Dunn post-hoc test of EZt50 and MZt50, where the experimental (blue) genotype was significantly different from both control (brown, green) genotypes. EZt50 and MZt50 allow normalisation of the total levels of activity among genotypes.

In spite of the disappointing nature of the results described above, we used dsRNAi to knockdown *norpA* in clock and clock-related neurons and examined any behavioural repercussions. We first confirmed using Western blots that the VDRC *UAS–norpARNAi* construct did indeed significantly and robustly reduce levels of NORPA, by driving it with *actinGal4* (Figure S3). We then crossed the UAS construct to *timGAL4,* which drives expression in all clock neurons in the brain and in the eyes, so we might expect that *norpA* downregulation should give a similar phenotype to the *norpA* null mutant, which increases *per 3′ UTR* splicing, leading to an advance in the evening (E) and morning (M) components of locomotor behaviour. As illustrated in Figure 4A, at 18 °C, there is a considerable advance in the evening EZt50 of locomotor activity compared to both controls (Kruskal–Wallis, *p* = 0.0002, Table S1 which shows all the statistical analyses for these experiments) but no differences at 29 °C (*p* = 0.02 from one control only, Figure 4B, Table S1). In contrast, the morning MZt50 was also advanced at the higher temperature (Figure 4B, *p* = 0.0002) but not at the lower one (Figure 4A, *p* = 0.34). We also utilised the *clk6-1Gal4* driver, which, like *timgal4,* expresses in all canonical clock neurons [58] and observed very similar results, with an advance in the EZt50 at 18 °C only (Figure 4C, *p* = 0.0006) and a similar advance in the MZt50 at warmer temperatures (*p* = 0.0006, Figure 4D). *norpA* is expressed at high levels in the eyes, where *timGAL4 and clk6-1Gal4* are also expressed, so we investigated whether the advance in locomotor behaviour might also have a contribution from the eyes. However, when we used the *gmrGal4* eye driver, we observed significant advances in the EZt50 at warmer temperatures only (*p* = 0.0001) and no differences in MZt50 (Figure 4E,F). These results suggested that the large advance in E behaviour driven by *timGal4* and *clk6-1Gal4* observed under colder conditions was due to *norpA* downregulation in clock neurons, not the eyes, but the latter nevertheless contribute substantially to the E advance reported in *norpA* mutants at warmer temperatures [51,52].

We next utilised the *PdfGal4* driver to manipulate *norpA* levels in the ‘morning’ (M) cells (s-LNvs and l-LNvs, Figure 4G,H). Surprisingly we observed a significant delay in the EZt50 (*p* = 0.0016) compared to both controls at low temperature and no significant effects for the MZt50 (Figure 4G). We also used the *Mai*^179^*Gal4* driver which expresses in a similar but wider pattern to *PdfGal4,* namely in the s-LNvs, a subset of l-LNvs but also in the CRY-positive LNds and weakly in a couple of DN1s [20] and saw similar effects, with a delay in the EZt50 at 18 °C (Figure 4I). However, statistically, this was not as dramatic as the delays with *PdfGal4*, in that it was significant against only one of the two parental controls, suggesting an amelioration of the delay because of the inclusion of additional neurons in the *Mai*^179^*Gal4* expression pattern (Figure 4I). Taken together with the *timGal4* and *clk1-6Gal4* results, the advance in the E locomotor component appears to be due to dorsally positioned clock neurons seemingly acting in opposition to the PDF cells and suppressing the LNvs-mediated delay. To study this further we used *timGal4*, *cryGal80* and *clk1-6Gal4*, and *cryGal80* to downregulate *norpA* only in CRY-negative cells, represented by half the DN1s, the two DN2s and almost all the DN3s and three of the LNds [27]. We observed no significant effects at all of *norpA* downregulation on the locomotor profiles compared to controls suggesting that it is the more dorsal CRY-positive clock neurons, rather than CRY-negative ones which are mediating these advances in locomotor components (Figure 4K–M).

Finally, we also downregulated *plc21C* using two RNAi lines from VDRC. We first checked that these VDRC *UAS* constructs downregulated *plc21C* with *actingal4* (Figure S4). Using RT-PCR, we observed that line 2 gave a generally more efficient knockdown at both 18 and 29 °C. Since NORPA and PLC21C share a 32% of homology in their primary amino acid sequence [62], it is possible that the *plc21C* RNAi may have off-target effects on *norpA* expression. To test this possibility, flies in which the downregulation of *plc21C* was driven by *actinGAL4* were used in Western blots to detect the level of NORPA. No significant differences between controls and experimental flies were detected (Figure S5). We next investigated whether the *plc21C* RNAi affected the splicing of the *per 3′ UTR.* We observed no significant changes in the *per* splicing pattern in the knockdown flies at either ZT0 or ZT12 (Figure S6). We also extended this analysis to the splicing of the *timeless^cold^* mRNA isoform [63] but again the downregulated flies did not show any changes in *tim* levels, nor the *tim* splicing pattern (Figure S7). Finally, we crossed the *UASRNAiplc21C* construct from line 2 to *timGal4*, *gmrGal4* and *PdfGal4* and examined the locomotor activity profiles. The values of EZt50 and MZt50 for the downregulated flies did not fall outside those for the two corresponding controls for any of the drivers (Figure S8, Table S1). Consequently, downregulation of *plc21C* does not appear to alter *per 3′ UTR* nor *tim^cold^* splicing, and nor does it lead to any of the *norpA* mutant-mediated phase advances on the phasing of the main M and E locomotor peaks at either warm or cold temperatures when driven in clock neurons.

## 4. Discussion

We have examined the effects of *norpA* downregulation in specific brain tissues in order to identify the anatomical focus for *norpA*-mediated enhancement of *per 3′ UTR* splicing that generates a significant advance in the circadian locomotor components at warm and cold temperatures [51,52]. We observe that the two pan-canonical clock neuron drivers, *timGal4* and *clk1-6Gal4*, generate consistent results, with both revealing the advances in the E component at colder and in the M component in warmer temperatures, recapitulating the effects reported for the *norpA^P^*^41^ mutant [51,52]. However, neither of these drivers generated the advance in the E component at higher temperature observed in *norpA* mutants. We therefore turned our attention to the *gmrGal4* driver that expresses strongly in the eyes and observed that downregulation of *norpA* led to the advance in the E component at high temperature. In contrast, the *PdfGal4* and *Mai*^179^*Gal4* drivers led to a delay in the E component at low temperature which was less pronounced with *Mai*^179^*Gal4,* suggesting that the CRY-positive LNds that are included in the latter driver’s expression pattern are partially suppressing the Pdf cell induced delay. Furthermore, downregulating *norpA* in the CRY-negative dorsal neurons (with *timGAL4*; *cryGAL80* and *clk1-6GAL4*; *cryGAL80*) had no effect, suggesting that it is the CRY-positive dorsal neurons (some DN1s and three LNds) that were mediating the low-temperature advances. These results are somewhat paralleled by those obtained under constant darkness in which the more dorsal clock neurons appear to have an endogenous shorter period that can inhibit the naturally longer free-running periods of the PDF expressing neurons [23]. Perhaps our LD experiment also uncovers this network property, albeit in a different context because the PDF cells are generating delays consistent with their longer periods, whereas the dorsal neurons are creating advances.

The CRY-positive dorsal neurons’ role in mediating the effects of *norpA* downregulation on *per* splicing and the resultant advances in the E behavioural component at low temperature is consistent with the absence of any temperature-dependent changes in locomotor activity in *cry^b^* mutants [51,52]. It also resonates with the findings that the E component requires PER expression in the LNds [20] and that ablation of CRY + PDF-neurons, which includes the LNds and some DNs also eliminates the E component [21]. The advance in the M component at higher temperature also appears to be due to CRY expressing dorsal neurons but this result is more difficult to understand given that the PDF cells are invoked to determine the M component. As well as 3 CRY-expressing LNds, at least 6–8 DN1 neurons express CRY and the group of 16 DN1 neurons has been invoked in both photic and thermal regulation of rhythmicity [64,65]. Furthermore, DN1s have inhibitory glutamatergic connections to the PDF and LNd cells that have been implicated in modulating the siesta and in the normal behavioural response to constant light [40,66]. However, at warm temperatures, the DN1s appear to reduce their neuronal output, so any influence on the PDF cells should be reduced [65]. It is therefore curious that *norpA* downregulation generates the M advance seen with *timgal4* and *clk1-6gal4* at high temperature. As the LNds are also included in this expression pattern, we suspect that they are also important in the M advance when *norpA* is downregulated. In this respect, although our in situ results were disappointing, a recent RNAseq study of the different clock neuronal groups revealed that the LNds expressed robust daily oscillations of *norpA* expression in LD cycles so it would appear that our method was not sensitive enough to detect *norpA* [67].

The delay in evening locomotor activity to the latter part of the day under hot, summer conditions suggests that it is an adaptive response, whereby flies avoid the desiccating effects of the hottest part of the day. Why the eyes might determine this process under summer conditions and the CRY-expressing subset of the dorsal neurons determine the winter phase is unclear. Two similar, if complicated, models of how *norpA* may activate an unknown suppressor of *per 3′ UTR* splicing under warn and cold conditions have been proposed [51,52]. Interestingly, based on the use of clock and visual mutants and their corresponding double mutants, the model by Collins et al. suggests that the visual system activates an unknown splicing repressor molecule during the day at high temperatures, which the clock maintains during the night [51]. The Majercak et al. model proposes that on hot days, the heat signal is transduced through *norpA*, so in mutants, less suppression of *per 3′ UTR* splicing occurs, leading to a colder phenotype and advance in the E component [52]. In this model, both clock-independent (visual) and clock-dependent pathways are invoked for splicing repression but in *norpA* mutants, these would be attenuated. If the clock-independent pathway is differentially more sensitive to the mutation at warmer temperatures, that might explain why the eyes determine the *norpA* phenotype under these conditions. Consequently, both models would appear to be consistent with the *gmrGal4* results described here.

The identity of the splicing repressor is unknown, but recently, using an S2 cell assay, *per 3′ UTR* splicing was observed to be decreased by the downregulation of serine/arginine-rich (SR) splicing factor B52 which binds to the *per 3*′ *UTR* [68]. Furthermore, polymorphisms in the *per 3′ UTR* that alter the levels of splicing [49] show changes in the binding affinity of B52 to these sites [68]. B52 was downregulated in the fly brain using three of the same drivers as in our study, *timGal4, PdfGal4* and *gmrGal4* at 18 °C. The phenotype assessed was sleep rather than locomotor activity, and downregulating *B52* using *timgal4* led to an increase in daytime sleep. *PdfGal4* driven *B52* downregulation led to a less dramatic enhancement of mid-day sleep, while *gmrGal4* had no effects [68]. While sleep is not simply an inversion of the locomotor activity profile, as B52 downregulation reduces *per 3*′ *UTR* splicing, whereas *norpA* downregulation enhances splicing, the results of Zhang et al. are not inconsistent with ours.

Finally, we also observed that downregulation of *plc21C* in clock neurons did not lead to any changes in *per* or *tim* splicing patterns nor did it generate the advance in the E locomotor component that is observed with *norpA* downregulation. Consequently, it would appear that Rh1/Rh5/Rh6 in the eyes and Rh7, which is expressed in PDF neurons and whose signalling is mediated by PLC21C, are not required for *3′ per UTR* splicing. If we add the finding that *norpA* downregulation in the eye did not give a significant advance in the E component at 18 °C, then we can largely rule out a role of the relevant eye-expressed rhodopsins in thermo-sensitive *per* splicing under these conditions. However, under warmer summer conditions, the photoreceptors which express CRY and the opsins—R1–6 and R8—are evidently recruited to the adaptive delay in locomotor activity, which is attenuated by the *norpA* mutation.

## 5. Conclusions

*D. melanogaster* show an adaptive response to warm temperatures in which the fly delays the evening locomotor component in order to avoid the hottest parts of the day. This response is mediated by splicing of a 3′ intron in the *per* gene, and can be altered by mutation in *norpA* which changes the *per* splicing pattern to generate a more ‘cold-like’ behavioural phenotype in which the evening (and morning) component is phase advanced. We have performed a preliminary neurogenetic dissection of the *per*-expressing neurons in which this splicing takes place by knocking down *norpA* in the relevant clock neurons. Our main results suggests that the eyes, in which both *per* and *norpA* are expressed, generates the advance in the evening component at warm temperatures and that the CRY-positive dorsal neurons (some DN1s and three LNds) appeared to be responsible for the *norpA*-mediated advance at cold temperatures. These results imply that environmental changes alter the influence of different components of the neuronal network that generates rhythmic behaviour and that the eyes, as well as the canonical clock neurons, play a prominent role.

## Supplementary Materials

The following are available online at https://www.mdpi.com/2079-7737/9/6/130/s1, Table S1: Results of Kruskall Wallis ANOVA. Supplementary experimental methods. Figure S1: NORPAand PDF ICC in *R32* brain. Figure S2: NORPA is expressed in the optic lobes. Figure S3: *norpA* knockdown with *actinGAL4*; Figure S4: Downregulation of *plc21C* mRNA at ZT0 and ZT12. Figure S5: *plc21C* downregulation does not alter NORPA levels. Figure S6: *plc21C* RNAi does not alter *per 3′UTR splicing.* Figure S7: *plc21C* knockdown does not affect *tim* levels or *timcold* splicing. Figure S8: Effects on locomotor activity of knockdown of *plc21C*.
