# Supplementary material for: Norpa Signalling and the Seasonal Circadian Locomotor Phenotype in Drosophila
Source: Biology (Basel). 2020 Jun 16;9(6):130. doi: 10.3390/biology9060130 (PMC7345481; doi:10.3390/biology9060130)
Supplement: Supplementary file 1 [file biology-09-00130-s001.pdf]

Supplementary Materials:

Norpa Signalling and the Seasonal Circadian Locomotor Phenotype in *Drosophila*

Carlo Breda, Ezio Rosato and Charalambos P. Kyriacou

Table S1. Primary and secondary antibodies used for immunocytochemistry.

Antibody	Source	Animal in Which It Was Raised	Fluorophore	Working Concentration
α -NORPA	Gift from Prof. Rouyer	Rabbit		1:1000
α -LAC	ABCam Ltd	Mouse		1:1000
α-PDF	Developmental Studies Hybridoma Bank	Mouse		1:600
α-GFP	ABCam Ltd	Rabbit/Mouse		1:1500
α -Rabbit	ABCam Ltd	Goat	Cy2	1:500
α -Mouse	Sigma-Aldrich	Goat	Cy5	1:500

Table S2. Results of Kruskall Wallis ANOVA and Dunn *posthoc* tests among the experimental (*Gal4/Gal80 driver x UAS-RNAi*) and control parental genotypes (driver or UAS-RNAi) crossed to w^{1118}. The significant *post hoc* results are highlighted in blue cells (advance) or red cells (delay). When the experimental group is significantly different from both control genotypes in a consistent direction (advance or delay), both cells are the same colour.

**18 °C**		**EZt50**			MZt50	
**Genotype**	**KW (H, p)**	***v UAS (p)***	***v Gal4/80 (p)***	KW (H, p)	*v UAS (p)*	*v Gal4/Gal80 (p)*
UASnorpAi						
timGal4	17.2 (0.0002)	0.002	0.0006	2.1 (0.35)	-	-
timGal4;cryGal80	14.24 (0.0008)	0.002	1	0 (1)	-	-
gmrGal4	5.4 (0.068)	-	-	0 (1)	-	-
PdfGal4	12.86 (0.0016)	0.003	0.03	0 (1)	-	-
Mai^179Gal4	14.83 (0.0016)	0.28	0.0005	0 (1)	-	-
clk6-1Gal4	14.74 (0.0006)	0.04	0.0012	0 (1)	-	-
Clk6-1Gal4;cryGal80	14.96 (0.0006)	0.145	0.0005	0 (1)	-	-
UASplc21Ci						
timGal4	5.0 (0.08)	-	-	0 (1)	-	-
gmrGal4	16.12(0.0003)	0.014	0.0024	0 (1)	-	-
PdfGal4	12.04 (0.0024)	0.003	0.5	0 (1)	-	-
29°C						
UASnorpAi						
timGal4	8.02 (0.0182)	0.374	0.038	16.86 (0.0002)	0.395	0.00018

timGal4;cryGal80	10.36 (0.0056)	0.395	0.432	1.05 (0.59)	-	-
gmrGal4	19.8 (0.0001)	0.00009	0.015	20.65 (~0)	0.203	0.000031
PdfGal4	9.49 (0.0087)	0.43	0.60	7.24 (0.027)	0.307	1
Mai^{179}*Gal4*	21.84 (0.0016)	0.00003	0.145	0.37 (1)	-	-
clk6-1Gal4	7.31 (0.026)	1	0.52	14.69 (0.0006)	0.026	0.0008
Clk6-1Gal4;cryGal80	24.71 (~0)	0.033	0.097	7.43 (0.024)	0.072	1
UASplc21Ci						
timGal4	1.89 (0.39)	-	-	20.67 (~0)	0.74	0.01
gmrGal4	33.39 (~0)	0.011	0.03	17.52 (0.0002)	0.067	0.39
PdfGal4	22.13 (~0)	0.002	1	8.84 (0.012)	1	0.07

Supplementary information

In Situ Protocol

Initially, *norpA* expression has been evaluated following Wülbeck and Helfrich-Förster protocol (2007). *w1118* cDNA was used as template from which the *norpA* probe was synthesised by a nested PCR strategy. First a large region that included the template for the *norpA* probe was amplified and used as a source of DNA for the subsequent PCR reactions. These were carried out utilising two sets of primers that include in their sequence either T7 or T3 RNA polymerase promoters. The product of this PCR was subjected to RNA transcription in order to obtain a sense *norpA* probe and an anti-sense *norpA* probe (Figure 3.11). In both probes, a ribonucleotide conjugated with Digoxigenin (DIG) was incorporated in the reaction in order to be recognised by a specific antibody in the following *in situ* steps.

Brains were rehydrated through downgrading 90%, 70%, 50%, 30% methanol/0.2% PBT series (1× PBS plus 0.2% Triton-X) and finally washed for five times in PBT. Brains were then treated with proteinase K in order to increase the probe penetration for 3 min at room temperature and subsequently for 1 h at 4 °C. Proteinase K treatment was concluded by three washes with glycine (10 mg/mL) and subjecting brains to 4% paraformaldehyde in PBS fixation. After being washed for 5 times in PBT, samples were incubated for 5 min in a 1:1 mixture of PBT:HYB (50% formamide, 5X SSC-DEPC, 0.1% Triton-X, 100 μg/mL heparin, 100 μg/mL salmon sperm DNA and 500 μg/mL of yeast t-RNA) at room temperature. This mix was replaced with HYB and brains were incubated for 2h at 60°C. Subsequently, samples were incubated with serial dilutions (50, 100 or 150 ng) of RNA probe dissolved in HYB buffer at 60 °C ON. After overnight hybridisation, the excess of probe was removed through two downgrading washes: the first of 3:2, 1:1, 2:3 ratio of HB:2XSSCT at 60 °C (10× SSC: 3 M NaCl, 0.3 M sodium citrate, DEPC H2O) and the second with a 3:2, 1:1, 2:3 ratio of 0.2× SSCT:PBT at room temperature. Subsequently brains were washed three times with TNT (0.1 M Tris HCl pH 7.5, 0.15 M NaCl and 0.05% Tween 20) before being incubated for 2 h in TNB (0.5% Blocking Buffer supplied with Tyramide Detection Kit dissolved in TNT). Brains were then incubated ON with antibody diluted in TNT (dilution 1:100). Two different set of antibody, which recognise the modified nucleotide included during the RNA probe synthesis, have been used separately: one with horse-radish peroxidase epitope and another one conjugated with biotin. In the case of this latter, a secondary antibody has been used which recognises biotin and it is conjugated with horseradish peroxidise. In both cases, the luminescence signal was generated by the peroxidase which cleaves the Tyramide molecules. In order to remove the excess unbound antibody, brains were washed five times in TNT for 15 min each and, subsequently, incubated for 2-3 h in a solution containing Tyramide (Cyanine 3, diluted 1:50 in amplification buffer). At the end of the detection reaction, brains were washed three times with TNT for 15 min each and mounted in a slide using Vectashield to amplify

and protect the fluorophore signal. The samples were further processed to detect PDF protein or LACZ. In this case, brains were treated with TNB buffer and the ICC protocol was followed.

Primers Used for In Situ

Primer	DNA sequence
**NRPext F**	*5′ - TGTACCACAAGGTGTGTCCCC - 3′*
**NRPext R**	*5′ - TTCTCCTGGCGCAGAGATTC - 3′*
**T3NRPF I**	*5′- AATTA**ACCCT**CACTAAA**G**GGAGAACGGAGAAATTGGTGTACACG - 3′*
**T7NRPR I**	*5′ - TAATA**CGACT**CACTATA**G**GGAGATGATCTGGTAGCGGCTCC - 3′*
**T3NRPF II**	*5′ - AATTA**ACCCT**CACTAAA**G**GGAGAACCAGATCATCCGCTGGATC - 3′*
**T7NRPR II**	*5′ - TAATA**CGACT**CACTATA**G**GGAGATGCCCACTTTCTTGTCGGA - 3′*
**T3NRPF III**	*5′ - AATTA**ACCCT**CACTAAA**G**GGAGATTGATCCGTTTGCCGATG - 3′*
**T7NRPR III**	*5′ - TAATA**CGACT**CACTATA**G**GGAGAGGTCTTTTGCACCGAGGTG - 3′*
**T3NRPF IV**	*5′ - AATTA**ACCCT**CACTAAA**G**GGAGAAGATGCTGGCAAGGCCAA - 3′*
**T7NRPR IV**	*5′ - TAATA**CGACT**CACTATA**G**GGAGACGCCTCCTCCTCGATTTTG -3′*

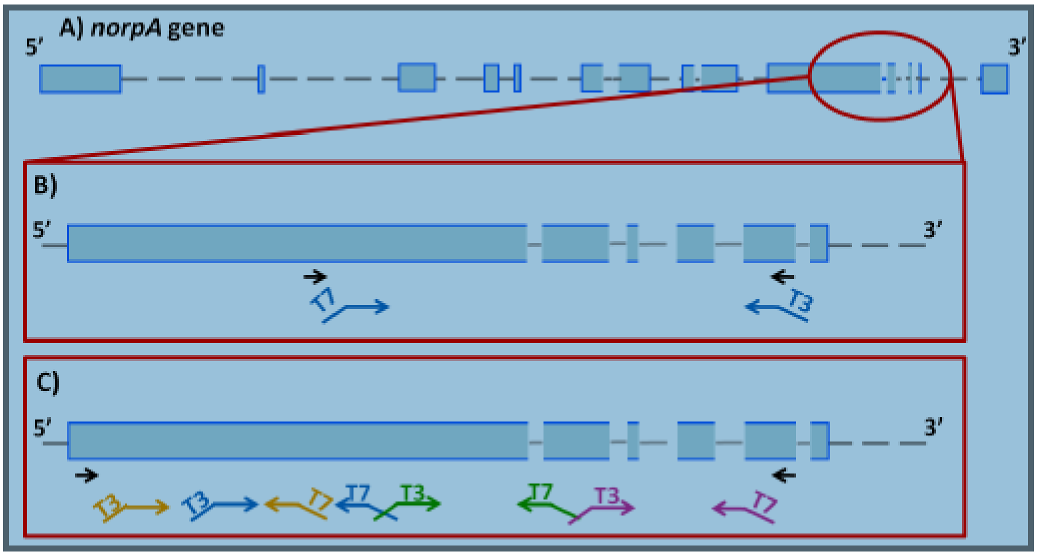

Strategy Adopted to Generate the Sense and Anti-Sense Probes Used for the In Situ Hybridisation

A) full length *norpA* is reported including exons and introns. B and C), an enlargement of the region amplified for generating the probes is shown. Black arrows indicate the first set of primers utilised for amplifying a larger region that has been used as template for the following PCR reactions using the T7 and T3 probes (different coloured arrows).

Immunocytochemistry in Drosophila Brain (ICC)

Dissected brains were washed for 5 min in 75% MetOH in PBT (PBS plus 0.5% of Triton-X). Two subsequent 5 min washes were performed with 50% and 25% MetOH in PBT, respectively, and finally three 5 min washes with 0.5% PBT in order to be permeabilised for the following steps. Subsequently, dissected brains were blocked for 2 h with 1% Bovine Serum Albumin (BSA) to minimise non-specific binding of the primary antibody. After blocking, the brains were incubated with the primary antibody which was diluted in 1% PBT which also contained 1% BSA and 0.1% Sodium azide. The presence of sodium azide prevents the contamination of the antibody solution with bacteria and thereby allows the re-use of the antibody. The brains were incubated in primary antibody for the required period of time (between 1 and 3 days) at 4 °C every day mixing by pipetting the solution. They were then washed five times for 5 min each in 1% PBT. After being washed the brains were incubated for 2 h at room temperature in the appropriate secondary antibody (raised again the animal

in which the primary antibody was generated) which was conjugated with a fluorophore. Once the protein of interest had been labelled with the appropriate antibody, the brain was mounted onto slides (VWR) with a drop of mounting medium which comprises of 80% glycerol and 3% propyl gallate and covered with a coverslip of 0.1 mm thickness (VWR). The slides were stored in the dark at 4 °C. They were visualised on an Olympus FV1000 confocal microscope. Individual images were taken of planes at different depths in order to create a Z-series for each brain. The size of the sections forming a Z-series was either 0.44 μM (if using 40× objective) or 1.4 μM (if using 20× objective). The optimal microscopic settings, in particular the laser gain, amplifier gain and offset and laser intensity were adjusted for each experiment in order to maximise the quality of the images. Dissected larval brains were treated similarly except that 3rd instar larvae maintained at 18 °C.

Primers Used for RT-PCR of plc21C

Primer	DNA sequence and numbering	Reference
plc21C F	*5′ - CCGGCCAGAAATCCAGTC - 3′*	FlyBase IDFBgn0004611
plc21C R	*5′ - CGATGCTGTTGTGCGACTT - 3′*	FlyBase IDFBgn0004611
rp49 F	*5′ - ATCGGTTACGGATCGAACAA - 3′*	FlyBase IDFBgn0002626
rp49 R	*5′ - GACAATCTCCTTGCGCTTCT - 3′*	FlyBase IDFBgn0002626

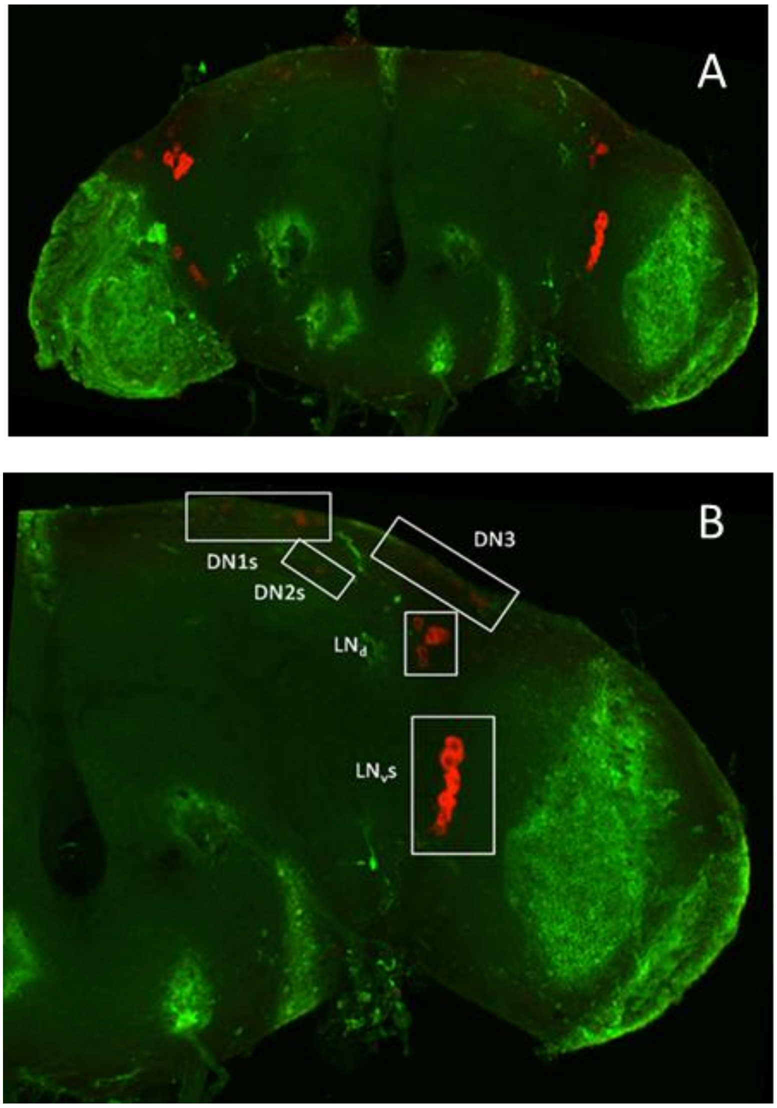

Figure S1. NORPA and PER-reporter expression. Representative *R32* Drosophila brain (20×, **A**) and enlargement of the right hemisphere (**B**). Confocal images were merged together in order to create a full tri-dimensional structure of the brain. The red signal (Cy5) corresponds to lacZ (PER reporter) whereas green (Cy2) identifies NORPA expression.

A

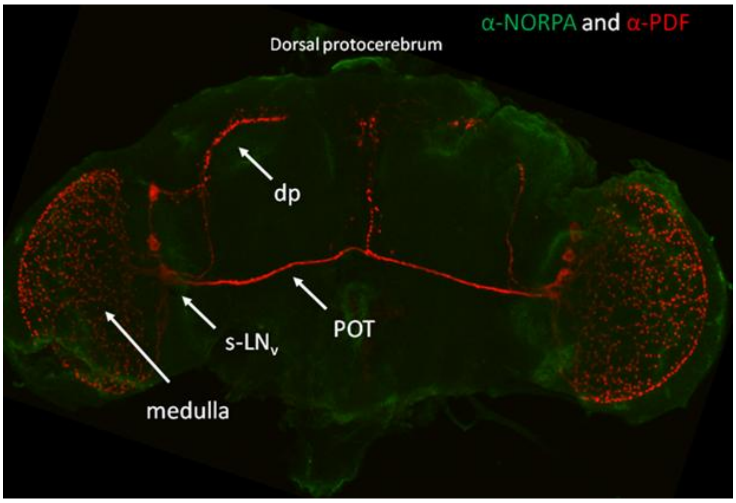

B

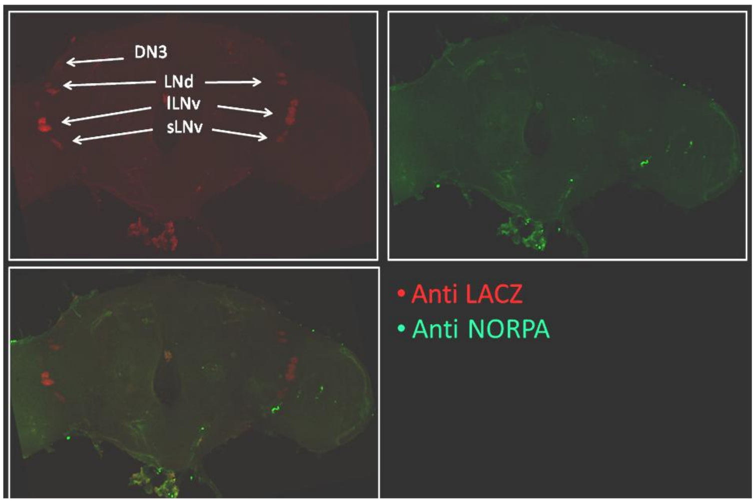

Figure S2. NORPA is expressed in the optic lobes. (**A**). *w*^{1118} brain labelled with PDF (red; Cy5) and NORPA (green; Cy2) antibodies. The brain presented is an image of 30 independent layers merged together. (**B**). *norpAp41*; *R32* adult brain. Confocal images were merged together in order to create a full tridimensional structure of the brain. The red signal (Cy5, in the top left panel) corresponds to LACZ antibody whereas green signal (Cy2, right top panel) represents NORPA antibody.

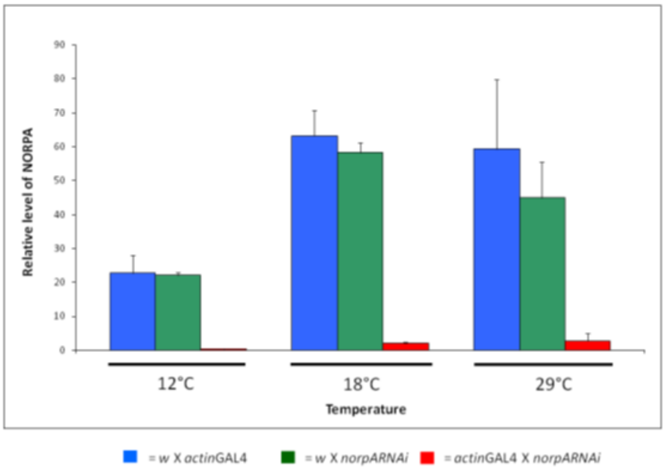

Figure S3. *norpA* knockdown with *actinGAL4*. Means +/- sems for 3 replicates are shown for 3 temperatures. Red is the experimental genotype, blue/green controls.

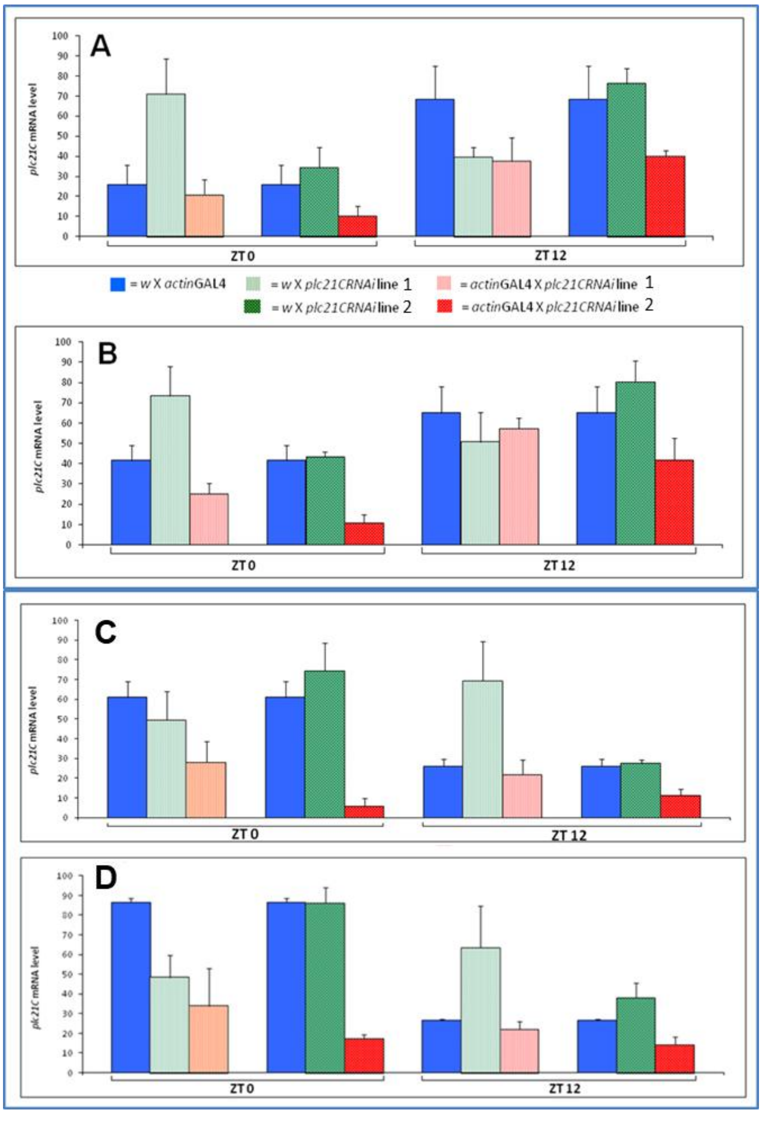

Figure S4. Downregulation of *plc21C* mRNA at ZT0 and ZT12. Panel A, B 18 °C; C, D 29 °C. **A, C** silencing within the region where the interference construct has been designed. **B, D** downregulation assessed in an external region upstream the interference region. Means ± sem for three independent replicates for lines 1 and 2.

A

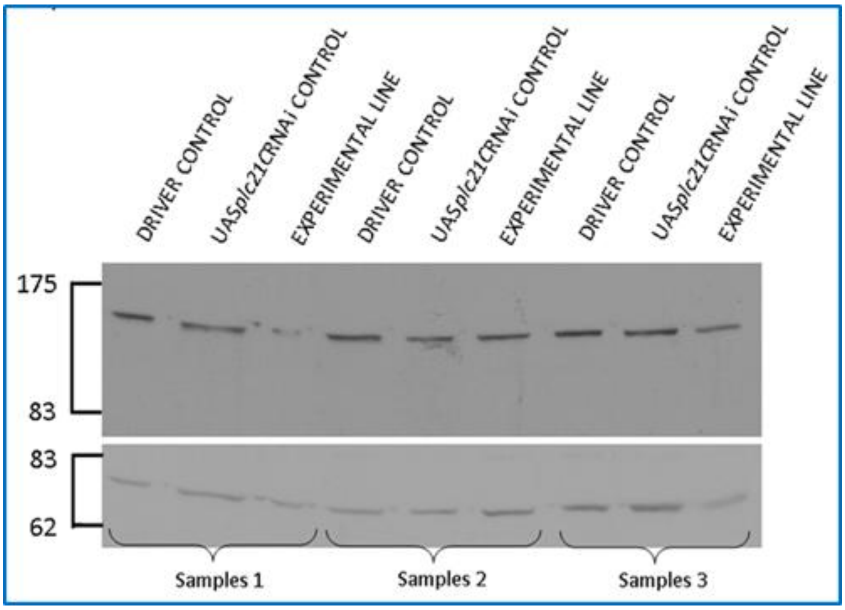

B

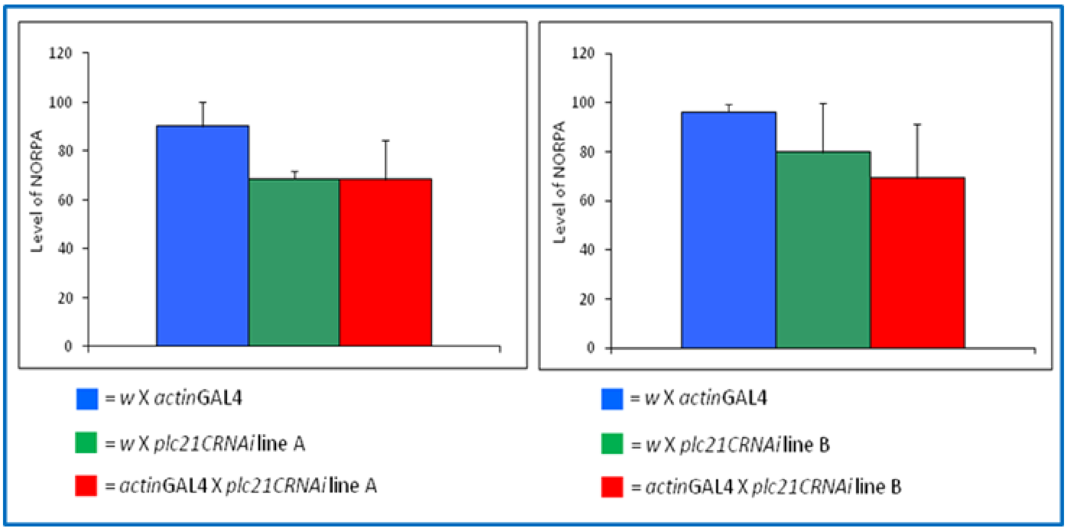

Figure S5. *plc21C* downregulation does not alter NORPA levels. (**A**) Western blots of NORPA in flies downregulating *plc21C* (line 2) and their controls (N = 3). HSP70 was used as a loading control. Lanes 1, 4 and 7 are driver controls (*w*; +; *actin*GAL4/+); lanes 2, 5 and 8 are RNAi controls (*w*; *plc21*CRNAi/+; +) and lanes 3, 6 and 9 *plc21C RNAi* flies (*w*; +; *actin*GAL4/*plc21CRNAi*). (**B**) Level of NORPA in flies downregulating PLC21C in line 1, and C, line 2. Means ± sem. For each gel the most intense band was set equal to 100 and the others were normalised accordingly.

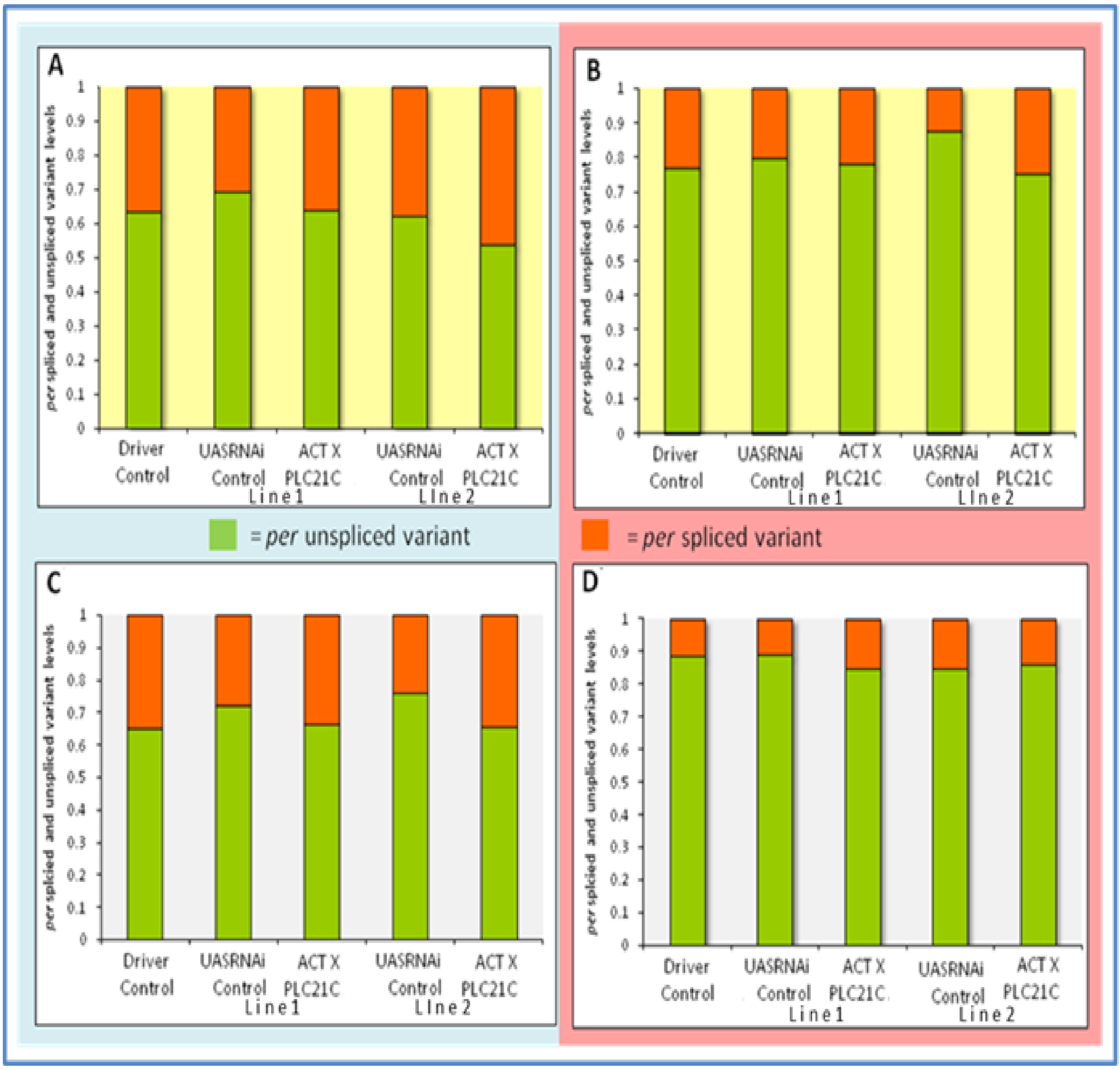

Figure S6. *plc21C* RNAi does not alter *per 3′UTR splicing*. Levels of spliced and unspliced *per* variants in *actin*GAL4 X *plc21C* RNAi lines 1 and 2 at 18°C (**A** and **C**) and 29°C (**B** and **D**). ZT 0 (**A** and **B**), ZT 12 (**C** and **D**).

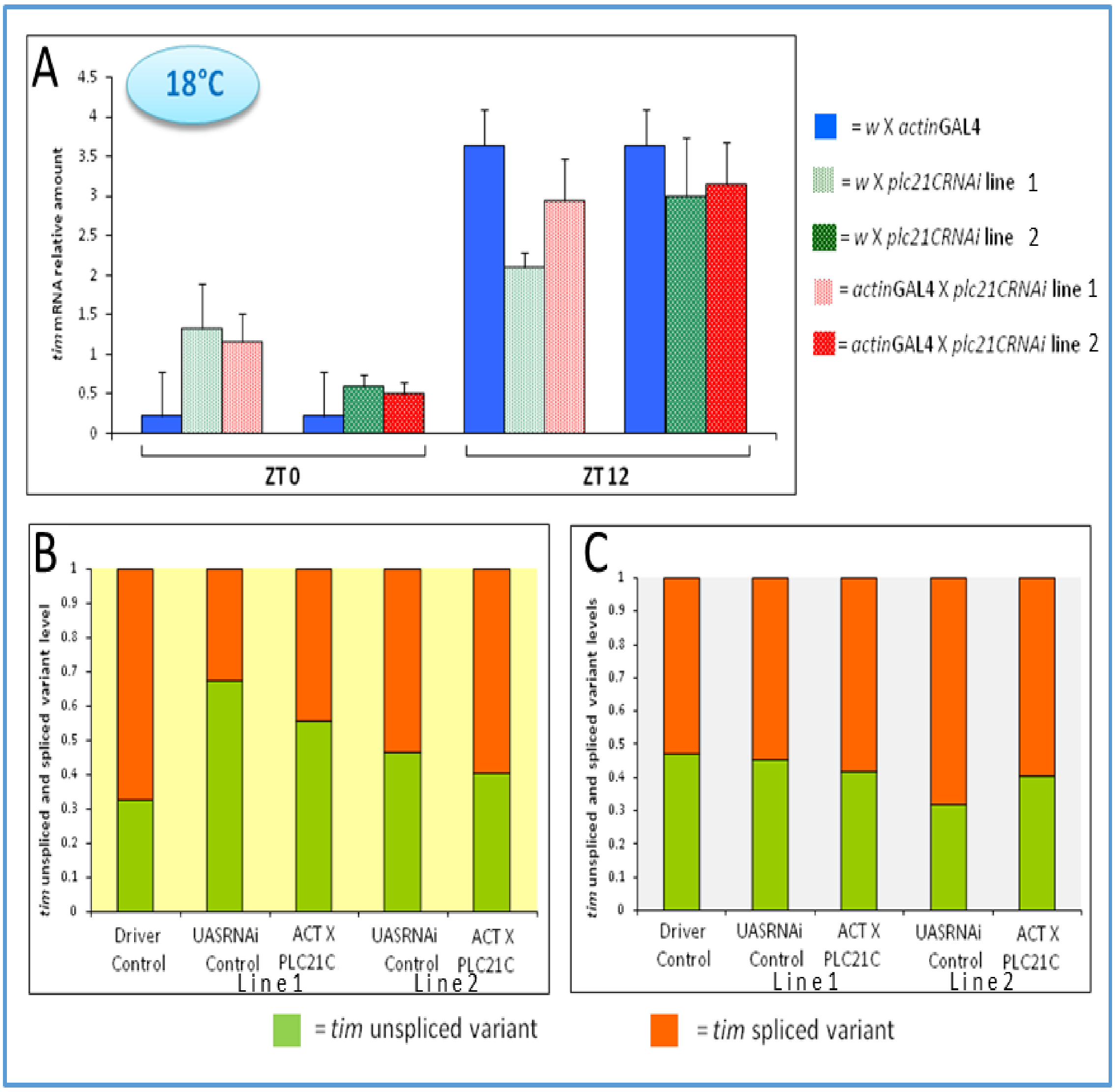

Figure S7. *plc21C* knockdown does not affect *tim* levels or *tim*^{cold} splicing. (**A**). Total *tim* level at ZT 0 and ZT 12 in *actinGAL4* x *plc21C* RNAi flies at 18°C. (**B**) and (**C**) show the unspliced and spliced levels at ZT 0 and ZT 12, respectively.

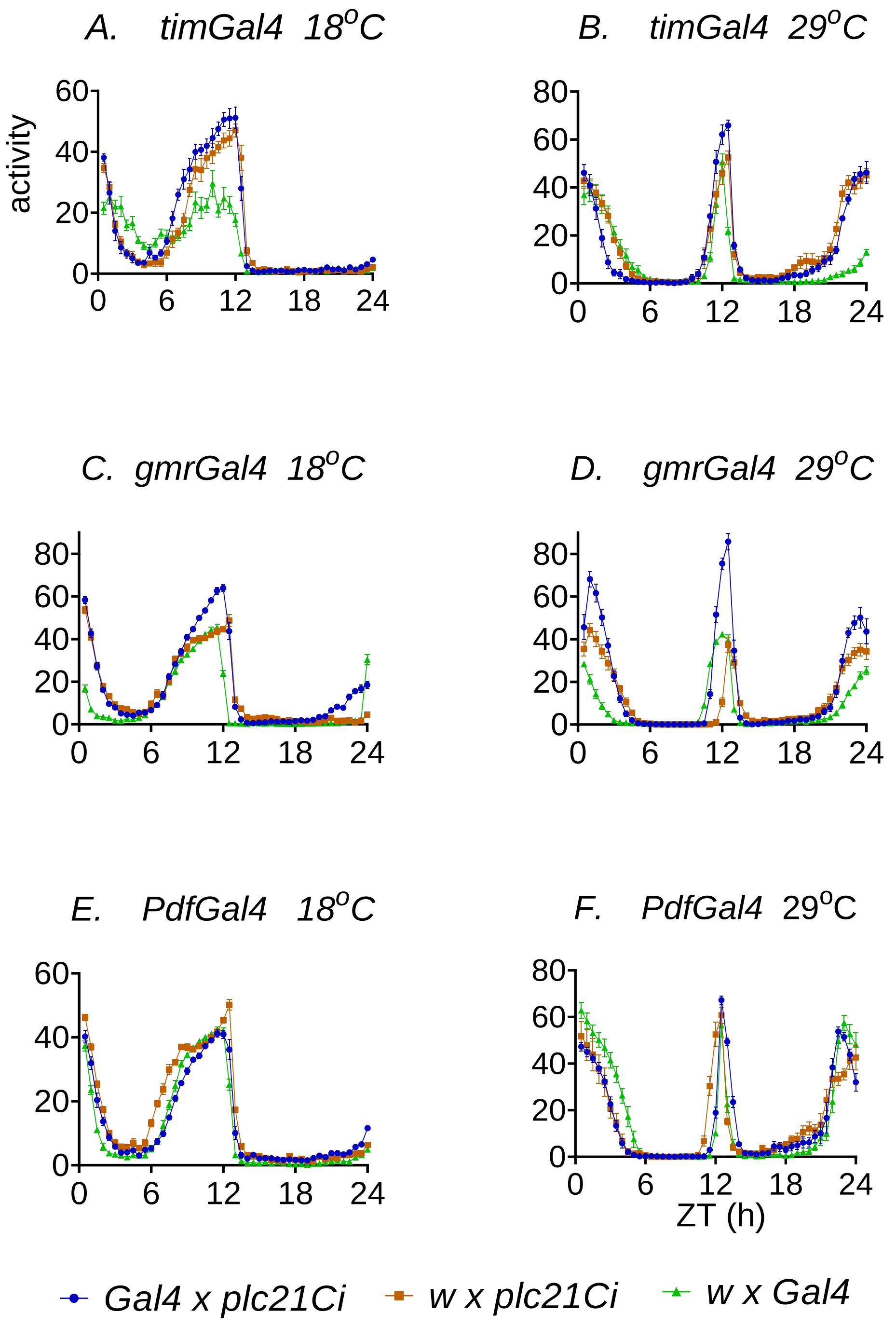

Figure S8. Effects on locomotor activity of knockdown of *plc21C*. Mean (+/- sem) locomotor activity events per 30 min time bin are plotted against ZT in an LD12:12 schedule, ZT0-12 day, ZT12-24 dark. Blue trace is the experimental genotype (*Gal4 x plcCi*), brown is *w x UASplcCi*, and green is *w x Gal4*. Kruskall Wallis ANOVA followed by Dunn *a posteriori* procedure was applied to EZt50 and MZt50 values. There were no examples where the experimental (blue) genotype was significantly different from both controls.
